# Obtention and Study of Polyurethane-Based Active Packaging with Curcumin and/or Chitosan Additives for Fruits and Vegetables—Part I: Analysis of Morphological, Mechanical, Barrier, and Migration Properties

**DOI:** 10.3390/polym15224456

**Published:** 2023-11-18

**Authors:** David Ruiz, Yomaira L. Uscátegui, Luis Diaz, Rodinson R. Arrieta-Pérez, José A. Gómez-Tejedor, Manuel F. Valero

**Affiliations:** 1 Energy, Materials and Environment Group GEMA, School of Engineering, Universidad de La Sabana, Campus del Puente del Común, Km. 7, Autopista Norte de Bogotá., Chía 140013, Colombia; davidruga@unisabana.edu.co (D.R.); yomaira.uscategui1@unisabana.edu.co (Y.L.U.); rodinson.arrieta@unisabana.edu.co (R.R.A.-P.); manuelvv@unisabana.edu.co (M.F.V.); 2 Bioprospecting Research Group, School of Engineering, Universidad de La Sabana, Campus del Puente del Común, Km. 7, Autopista Norte de Bogotá., Chía 140013, Colombia; 3 Centre for Biomaterials and Tissue Engineering, Universitat Politècnica de València, 46022 Valencia, Spain; jogomez@fis.upv.es; 4 Biomedical Research Networking Centre in Bioengineering, Biomaterials and Nanomedicine (CIBER-BBN), 46022 Valencia, Spain

**Keywords:** chitosan, curcumin, food packaging, overall migration, oxygen transmission rate, polyurethane, water vapor transmission rate

## Abstract

Several polyurethane-formulated films with curcumin and/or chitosan additives for food packaging have been previously obtained. The study examines the effect of the additives on the film’s morphological, mechanical, barrier, and migration properties. Fourier-transform infrared spectroscopy (FTIR), scanning electron microscopy (SEM), water contact angle, thermogravimetric and differential thermal analysis (TGA and DTGA), differential scanning calorimetry (DSC), dynamic mechanical thermal analysis (DMTA), oxygen transmission rate (OTR), water vapor transmission rate (WVTR), and the overall and specific migration tests were conducted. The results show that the presence of chitosan significantly increased the overall migration and mechanical properties, such as the elongation at break, tensile strength, and Young’s modulus of most polyurethane formulations, while curcumin had a minor influence on the mechanical performance. Based on the results, formulations with curcumin but without chitosan are suitable for food packaging.

## 1. Introduction

Extending the shelf-life of produce is one of the main objectives of food packaging by protecting the product against external physical, chemical, and microbiological surrounding effects: for instance, moisture, gases, odors, microorganisms, and forces [1]. Hence, the main properties to consider in materials for food packaging are mechanical (such as elongation at break and tensile strength), thermal performance, barrier properties (i.e., water vapor transmission rate (WVTR) and oxygen transmission rate (OTR)), and overall migration. The properties of a material depend on the chemical structure of polymers and the intermolecular forces among polymer molecules and additives. A material intended for food packaging should have proper mechanical and thermal properties in order to keep a barrier between the product and the environment. It also should reduce the transmission of water vapor to decrease the loss water of the produce. The transmission of oxygen should be low to decrease the respiration of the food but not too low to avoid the anerobic atmosphere, which increases the production of ethylene. The migration from material in contact with food should be as low as possible. This is one of the most important criteria for the safety of these materials. The maximum overall migration allowed is 50 mg/kg according to the Colombian legislation or 60 mg/kg according to the European Union legislation [2,3].

Most of the materials used for food packaging are full-synthetic petroleum-based polymers due to their good mechanical properties, feasible manufacturing processes, good migration and barrier properties, and high transparency [4]. In that sense, the most used material for fruit and vegetable packaging is polyvinyl chloride (PVC) [5]. However, most of the current PVC food packaging films lack antimicrobial or antioxidant activities that might extend the shelf-life of produce [6,7].

Polyurethanes are a potential alternative material to PVC food packaging films [8,9]. Polyurethanes (PUs) are one of the most used plastics around the world due to their application in a wide array of technological fields, such as coatings, adhesives, paints, foams, wound dressing, drug delivery, films, and food packaging [10]. PU is one of the most versatile materials as well because of the extensive combinational possibilities for their synthesis conferred by the chemistry of polyurethanes [11,12,13]. Also, it is possible to functionalize polyurethanes through the addition of compounds with bioactivity to reach a useful interaction between packaging, environment, and food [14,15]. These functionalized materials could extend the shelf-life of food, since the microbial growth and oxidation of products are some common causes of spoilage [5,16]. Additives, like chitosan and curcumin, could extend the shelf-life of food. Chitosan is a heteropolysaccharide that is often used as antibacterial agent [17]. Curcumin is a natural polyphenolic compound with antibacterial and antioxidant activity [18,19].

Several polyurethane formulations have been created with or without additives; most of the additives in PU formulations for food packaging are intended to confer antimicrobial or antioxidant properties [20,21,22]. Turan et al. proposed polyurethanes based on castor oil, polyethylene glycol (PEG), butanediol, and isophorone diisocyanate without additives. They found that increasing the molecular weight of PEG enhanced the thermal stability, oxygen permeability, and hydrophilicity, but it decreased the WVTR and mechanical properties [23,24,25]. Sarojini et al. shaped a film based on chitosan and polyurethane with mahua oil as polyol. They included nanoparticles of ZnO as an additive. They found that increasing the ratio of polyurethane without ZnO reduced the WVTR, oxygen permeability, hydrophobicity, and mechanical properties, but adding the ZnO additive improved those properties. They also found that a high ratio of ZnO particles improved the antibacterial activity as well as the shelf-life of carrots [26]. Zhang et al. created polyurethane with curcumin as the chain extender and glycerol as the plasticizer. They found that reducing the ratio of glycerol improved the UV shielding, water resistance, WVTR, oxygen permeability, and antioxidant activity, but it decreased the mechanical behavior [19].

Mulla et al. developed biodegradable films based on pectin obtained from the waste of apple juice and chitosan. They identified that chitosan and pectin in their pristine form lack thermo-mechanical properties. They combined chitosan and pectin to improve these properties. They found that the mechanical properties, transparency value, tensile strength, and elongation at break were significantly improved when they added pectin [27]. Kritchenkov et al. incorporated triazole betaine chitosan (TBC) into a succinyl chitosan sodium salt matrix (SC-Na); through the blend of TBC and SC-Na, they improved the antibacterial activity and tensile strength and reduced the oxygen and WVTR of the films. They carried out shelf-life tests on bananas and found that the film reduced the weight loss, vitamin C loss, and respiration rate, which extended the shelf-life of bananas [28]. Ji et al. prepared a chitosan film with micro-ramie fiber and lignin, reaching a significant improvement in tensile strength, mechanical, water resistance, and thermal and antioxidant properties. They applied shelf-life tests on chicken breast and tomato and found that they extended the shelf-life of chicken after 7 days of storage, but it did not extend the shelf-life of tomato due to the protection skin of this product. The authors found that the antibacterial ability of composite films reduced the spoilage of chicken [29].

Xiao et al. used chitosan nanoparticles incorporated with curcumin to improve the shelf-life of *Schizothorax prenanti surimi.* They found that at a chitosan concentration of 0.36%, the film had significantly extended the shelf-life of *S. prenanti* through antioxidant activity, leading to a reduction in weight loss, maintaining the pH and thiobarbituric acid reactive substances, and delaying physical and chemical changes, and it also reduced or inhibited the growth of microorganisms [30]. Yao et al. developed films of chitosan/poly (vinyl alcohol) reinforced with curcumin; they improved antioxidant activity, UV barrier property and tensile strength [31]. Li et al. prepared curcumin–chitosan film by encapsulation with dialdehyde starch. They reached films with good ultraviolet resistance and antioxidant capacity, and the films also had good antibacterial activity against *E. Coli* and *S. aureus*. They reduced the weight loss of apples, strawberries and mangoes after 7 days by 60%. [32]. O’Toole et al. conjugated curcumin and chitosan, and they improved the stability and solubility of curcumin, keeping its antioxidant activity [33]. Overall, those researchers identified the antibacterial and antioxidant activity of both chitosan and curcumin as well as the influence on the mechanical, physical and chemical properties of food packaging films. It highlights the poor solubility of curcumin and chitosan, which is a challenge for the processability of packaging films. As a counterpoint, these are both cheap and require low processability to obtain [18].

Despite there being several studies about the antimicrobial and antioxidant activities in food packaging polymers, the relation between chitosan of medium molecular weight and curcumin as additives in polyurethane films for food packaging has not been studied. The present work aims to obtain functionalized polyurethanes with chitosan and curcumin with antibacterial and antioxidant properties, characterize their morphological, mechanical, chemical, barrier, and overall and specific migration properties, and compare their performance with a film of commercial PVC. The matrix of PU was synthesized with castor oil, polyethylene glycol, and isophorone diisocyanate with chitosan and curcumin as additives. This study was divided into two parts. Part I studies effects of the physical and chemical interactions of chitosan and curcumin on the mechanical, morphological, barrier properties of the PU film and its subsequent food performance. Part II studies the antimicrobial and antioxidant activity as well as cell viability, degradability, and prolongation of shelf-life of some of the fruits and vegetables products.

## 2. Materials and Methods

### 2.1. Materials

Castor oil (CO) was purchased from Químicos Campota y Cía, Ltda., Bogota, Capital District, Colombia. Polyethylene glycol (PEG) with an average molecular weight of 1000 g mol^−1^ was purchased from Merck, Darmstadt, Hesse, Germany (CAS number 25322-68-3). Isophorone diisocyanate (IPDI) (CAS number 4098-71-9) was purchased from Sigma-Aldrich, Burlington, MA, USA. Chitosan medium molecular weight (CAS number 9012-76-4, 190,000 to 310,000 Da molecular weight) was obtained from Sigma-Aldrich, Burlington, USA. Curcumin (reference number 81025.1) was purchased from Cayman Chemical Company, Ann Arbor, MI, USA. PVC film was purchased at a local supermarket, Chia, Cundinamarca, Colombia.

### 2.2. Design of Experiments

We applied a design of experiments with two factors and three levels. The first factor was chitosan, comprising 0%, 1.5% and 3.0% (in weight) of the polyurethane matrix. The second factor was curcumin, comprising 0%, 0.25% and 0.5% (in weight) of the polyurethane matrix. The experiments were designed on Minitab Statistical Software 21.1.0, Minitab, LLC, State College, Pennsylvania, USA, in aleatory runs. Table 1 shows the order and code for each formulation.

### 2.3. Synthesis of Polyurethane Films

**Scheme 1:**
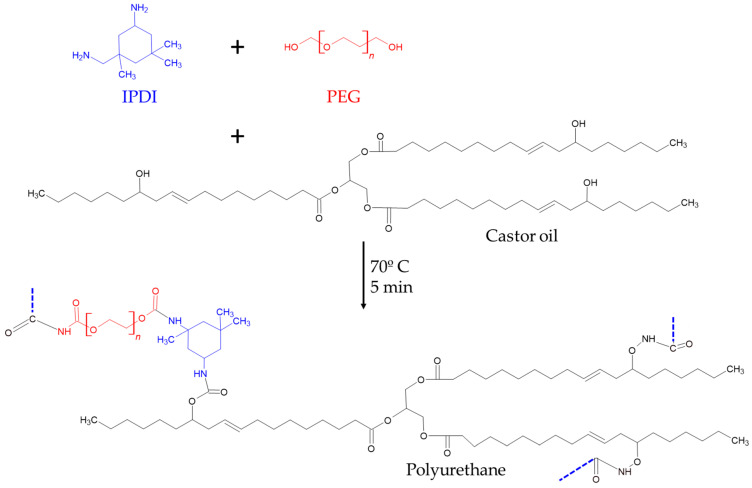
Reaction scheme of the polyurethane synthesis.

The polyurethane films were synthesized by the prepolymer method. First, we mixed the CO with curcumin in a beaker stirred at 200 rpm at room temperature for 10 min to resolve the solubility problems of curcumin. Second, we added PEG to the solution at 70 °C and stirred at 300 rpm. Third, we added IPDI at 70 °C. Fourth, we added chitosan, which was previously ground overnight, and then the solution was stirred at 300 rpm for five minutes. The polyurethane films were prepared through the method of casting-evaporation: the solution was poured into a sheet glass, an applicator of films was used to set the thickness at 0.5 mm, and then it was cured in an oven at 110 °C for 12 h. Scheme 1 shows a diagram of the polyurethane reaction.

### 2.4. Morphological Tests: Stethoscope and Scanning Electron Microscope (SEM)

We took SEM images of the surface and transversal cut of each PU film. We used an Olympus IX71, Center Valley, Pennsylvania, USA, equipment at 10×/0.4 for stethoscope analysis at 51×. SEM images were assessed with a desktop scanning electron microscope Tescan Lyra 3, operating at 6.0 kV voltage acceleration at 2.5 k× and a working distance of 10.85 mm. The films were cleaned with distilled water, and they were kept in a dried desiccator for 24 h. The surfaces of the samples were then treated with gold spray and observed using SEM. Afterwards, we assessed the superficial and transversal cut of each sample [34].

### 2.5. Color Measurement

Color coordinates were assessed at three random points on the surface of a cross-section of the film, following the procedure described by Dai et al. [35,36]. A CR-400 colorimeter, Konica Minolta Sensing Americas Inc., Ramsey, NJ, USA, was used as a D65 illuminating lamp and a 2° observer was used for measuring L* (lightness) a* (redness/greenness) and b* (yellowness/blueness).

### 2.6. Fourier Transform Infrared Spectra (FTIR)

The FTIR were recorded on a Nicolet iS10 FT-IR, Waltham, MA, USA, in the wave band from 400 to 4000 cm^−1^ by accumulating 64 scans at a resolution of 4 cm^−1^ [37]. FTIR spectra for films were recorded using an empty cell as the blank sample. To test the FTIR of curcumin and chitosan, KBr was used in a ratio of 1:50 *w*/*w*; and a sample of KBr pure was used for the control. The data were smoothed on Matlab R2022b version (MathWorks, Natick, MA, USA).

### 2.7. Thermal Stability: Thermogravimetric Analysis (TGA) and Differential Scanning Calorimetry (DSC)

The thermal performance of PUs was assessed in TGA and DSC equipment. The TGA was tested using a TGA/DSC Mettler Toledo Star 1 System, Greifensee, Uster, Switzerland, according to ASTM D6370 [38]. It tested thermal stability through onset temperature and differential thermogravimetric analysis (DGA). The heating rate was 10 °C min^−1^ in a temperature range of 25–600 °C, under a nitrogen atmosphere, testing 15 ± 2 mg samples [39]. The DSC was tested on a DSC 3+ Stare System, Mettler Toledo. The conditions were set up as follows: temperature in a range from −70 to 150 °C, nitrogen atmosphere with 20 mL min^−1^ flow and sample weights of 10 ± 2 mg [39].

### 2.8. Water Contact Angle

The surface hydrophobicity was measured by contact angle using a Drop shape analysis system GH11 Kruss, Germany, according to ASTM D7490-13 [40]. The assays were carried out by depositing a water drop of 10 µL ± 1 µL using a micrometer syringe. The contact angle reported is the median of ten tests on the same sample applied in different regions [39].

### 2.9. Dynamic Mechanical Thermal Analysis (DMTA)

The DMTA test was carried out using a DMA 850, Worcester County, MA, USA, with a tension mode at 1 Hz oscillation frequency, a deformation of 0.1% and a 0.02 mm displacement [41]. We applied a nitrogen purge at a rate of 60 mL min^−1^. The maximum in the damping peak of the tan(δ) was considered as the glass temperature (T_g_) [41]. The test was performed in triplicate with a sample size of 2 ± 0.1 cm × 0.5 ± 0.1 cm. The tests were evaluated on DMA 850 TA equipment.

### 2.10. Oxygen Transmission Rate (OTR)

The oxygen transmission rate (OTR) was carried out on an Oxtran model 2/21 MH Mocon, Minneapolis, Minnesota, USA. The test was carried out according to ASTM D3985-17 [42] 40 h before the test. The equipment was set up at 23 °C ± 2 °C and 50% ± 10% of relative humidity. The conditions to perform the test were set up at 23 °C ± 2 °C and 50% ± 5% of relative humidity. The sample had a hexagon form of 60 mm from side to side. We determined the thickness of the sample. The sample was placed on the equipment, and Apiezon T grease was used to waterproof it. The gradient of oxygen was set up at 20 mL min^−1^, the flow of the A, B and zero chambers were set up at 10 mL min^−1^, and a time of three hours was considered for the retrofit; then, it takes the measures on A and B cells for 30 min. It measured the OTR of the sample for 24 h. The tests were performed in duplicate for the sample.

### 2.11. Water Vapor Transmission Rate (WVTR)

The water vapor transmission rate (WVTR) was carried out in a chamber of temperature and humidity Angelantoni^®^, Massa Martana, Umbría, Italy, using calcium chloride as the desiccant. The test was carried out according to ASTM E96/E96M [43]. The equipment was set up at 23 °C ± 2 °C and 50% ± 10% of relative humidity for 40 h. The conditions to perform the test were set up at 23 °C ± 2 °C and 50% ± 5% of relative humidity. The sample had a diameter of 60 mm. We determined the thickness of the sample and applied a desiccant to avoid interference with environmental water. It was sieved in a diameter between 0.6 and 2.36 mm and dried at 200 °C. The chamber of equipment was set up at 38 °C ± 1 °C and 90 ± 2% of relative humidity. Then, we added 5 g of desiccant to the mold. The sample was placed on the equipment. We introduced the cover on the chamber, verifying that conditions of temperature and humidity were set up. We took the weight of the sample once per day and at the same hour ± 15 min. The test was performed in quadruplet.

### 2.12. Overall Migration

The global migration test was performed according to the UNE-EN 1186 method [44]. Cut samples of 5 cm × 5 cm were total totally immersed in 25 mL of acetic acid 3% *w*/*v* (simulant B used for fruits and vegetables) in distilled water solution, following the recommendation by UNE-EN 1186, which means 1 cm^2^ of film per 1 mL of simulant. The test was performed in 50 mL glass tubes. Both films and glass tubes were previously dried and weighted. The sample was placed in an incubator at 40 °C for 240 h. The polymer was removed, dried and weighted. The simulant was evaporated at 50 °C, and the solid residues on the vial were weighted in analytical balance to determine the overall migration as mg kg^−1^. The assay was carried out in quadruple. The overall migration mass and mass of empty vials were established by subsequent weights each after 24 h and stored in the desiccator until having a difference lower than 1 mg [45,46].

### 2.13. Specific Migration: Determination of IPDI, Chitosan and Curcumin

The specific migration was tested to the samples of overall migration. We measured the specific migration of isophorone diisocyanate (IPDI), chitosan and curcumin. We added 1 mL of formic acid 99% and 9 mL of dimethyl sulfoxide (DMSO) to the sample. Then, we applied ultrasound for 20 min. The sample was left in the water bath at 75 °C for 20 min. The samples were left at room temperature; then, they were filtered at 22 µm and drawn off to the equipment injection vial of 2 mL. The DMSO successfully dissolved IPDI and curcumin. This solvent was the working solvent, and it was used for the calibration curves [47,48,49,50]. To dissolve chitosan, it was hydrolyzed to reduce its molecular weight and assess the glucosamine monomer. It was weighted with 10 mg of chitosan; then, we added 3 mL of HCl (37%) and 0.5 mL of water type I. It was left in the water bath at 75 °C for 2 h. The volume was completed to 10 mL in order to have a standard pattern of 1000 mg L^−1^, which was used for the calibration curve [51]. Once the samples were filtered to the injection vials, they were assessed on a UPLC MS/MS Xevo TQD by Waters, Milford, MA, USA, with the column ACQUITY UPLC CSH C18 1.7 µm of 2.1 × 100 mm. The mobile phases were acetonitrile and water type I with 0.1% of formic acid [52].

### 2.14. Specific Migration: Determination of Molecular Weights

Once we determined the specific migration of IPDI, chitosan and curcumin, there are remaining undetermined masses. We determined the molecular weights of the remaining residues of obtained masses of overall migration in order to elucidate the possible other migrants. We extracted the analyte from the samples adding 1 mL of formic acid 99% and 9 mL of dimethyl sulfoxide. Then, we applied ultrasound for 20 min and submerged the samples in a water bath at 75 °C. Afterwards, they were filtered with a 22 µm filter and poured into injection vials. The samples were analyzed in a UPLC MS QTOF G2-XS Waters, Milford, MA, USA, with a column ACQUITY UPLC BEH C18 1.7 µm of 2.1 × 100 mm. The mobile phases were acetonitrile and water type 1 with 0.1% of formic acid. The data are shown in a ratio of monomers to the total overall migration. We reported the molecular weights of monomers through this method to determine whether the polymers migrated.

### 2.15. Statistical Analysis

The quantitative data outcomes were validated in the assumptions of normality of residues, homoscedasticity, and independence of variables. According to validation of assumptions, the data were analyzed through parametric or non-parametric statistics. The data of lightness (L*), water contact angle and overall migration were analyzed with a non-parametric Kruskal–Wallis’s test. The significant difference among groups was analyzed through Dunn’s test, using the median shown in the figures and tables. Data of redness/greenness (a*), yellowness/blueness (b*), Young’s modulus, tensile strength, elongation at break and WVTR were analyzed with ANOVA. The significant difference among groups was established through the Tukey test. The median of data is shown in the tables and figures.

## 3. Results and Discussion

### 3.1. Morphological Assays: Stereoscope and Scanning Electron Microscopy (SEM)

**Figure 1:**
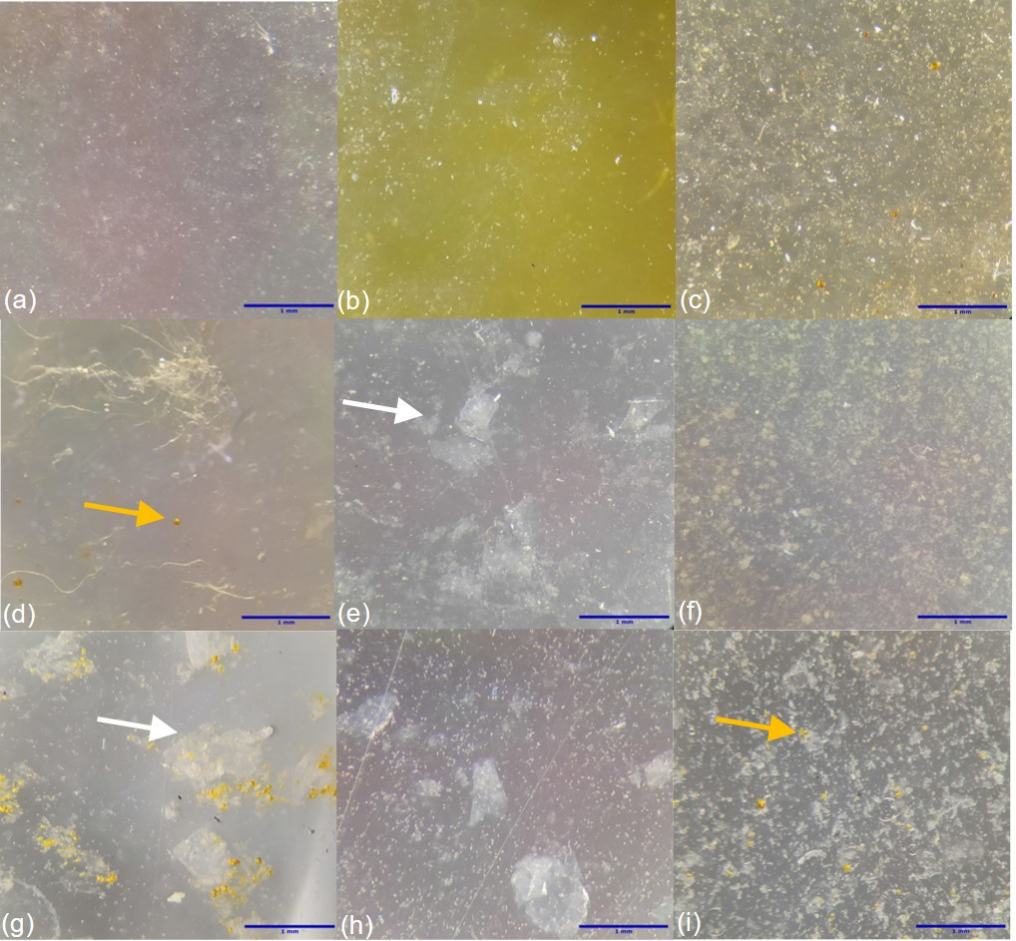
Stereoscope images of polyurethane formulations: (**a**) CH00CUR000, (**b**) CH00CUR050, (**c**) CH15CUR050, (**d**) CH00CUR025, (**e**) CH30CUR000, (**f**) CH15CUR025, (**g**) CH30CUR050, (**h**) CH15CUR000, and (**i**) CH30CUR025. The blue bar on the scale is equivalent to 1 mm. The white arrows point to chitosan, and the yellow arrows point to curcumin.

A stereoscope was used to verify the dispersion of the chitosan and curcumin additives in a horizontal plane of PU films. In Figure 1, the yellow points are curcumin particles (Figure 1b–d,f,g,i). The white points are chitosan granules (Figure 1c,e–i)). Figure 1 shows that curcumin and chitosan are dispersed on the polyurethane matrix, which means there is no evidence of aggregation.

**Figure 2:**
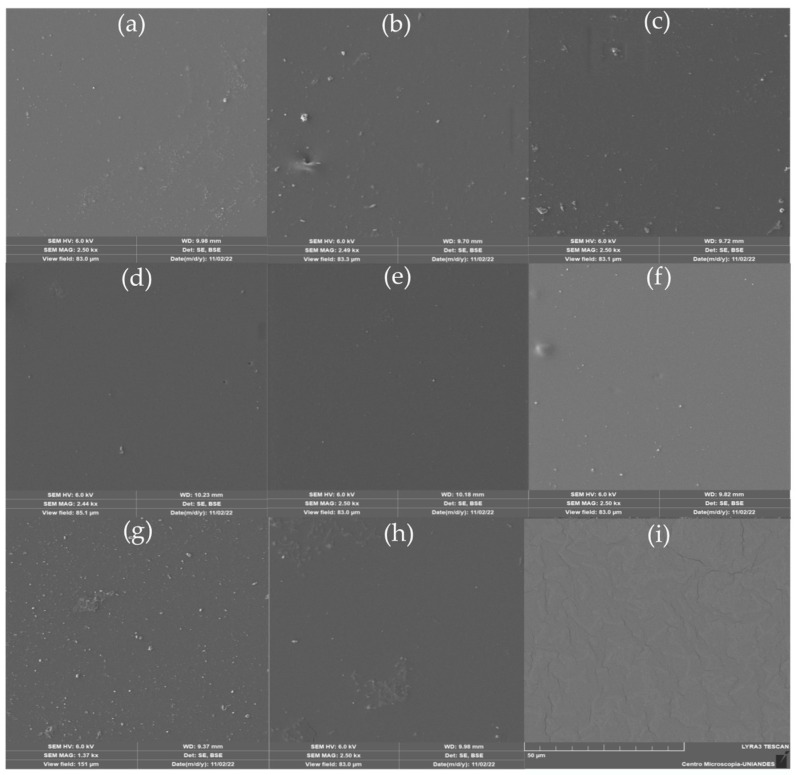
Superficial SEM images of PU formulations: (**a**) CH00CUR000, (**b**) CH00CUR050, (**c**) CH15CUR050, (**d**) CH00CUR025, (**e**) CH30CUR000, (**f**) CH15CUR025, (**g**) CH30CUR050, (**h**) CH15CUR000, and (**i**) CH30CUR025. The magnification of images is at 2.5 kx.

A homogenous smooth layer is observed on the surface images of all the PU films (Figure 2). Overall, all the SEM images of Figure 2 show the polyurethane matrix (dark zone) and the presence of additives particles that are both curcumin and/or chitosan. This reveals that the additives are not part of the polyurethane matrix; they are external agents of the matrix. As aforementioned, in all cases, the SEM images show a continuous phase (polyurethane matrix) and a dispersed phase (additives) without the presence of aggregates. Similar results were obtained by Sarojini et al. in polyurethane based on mahua oil and chitosan with different nanoparticles of ZnO [26]. The fact that additives are immersed in the polymer matrix is a desirable result that will prevent the additive from being washed with air, water, vapor or oxygen transmission between the food and the polymer films.

### 3.2. Color Analysis

The results of color analysis are shown in Table 2. The color analysis (lightness L*, redness/greenness a* and yellowness/blueness b*) was used as a control to show the polyurethane formulation without additives (CH00CUR000). We observed that the lightness L* axis decreases when the amount of additives increases (CUR and/or CH). This result is coherent with the images shown in Figure 1 due to it showing the dispersion of additives in the polyurethane matrix. However, there is no statistically significant difference between the control CH00CUR000 and the remaining materials with the exception of the CH30CUR000 formulation. On the other hand, we observed differences in both the redness/greenness (a*) and yellowness/blueness (b*) between the control (CH00CUR000) and remaining formulations with the exception of CH15CUR000. When the parameters a* and b* increased to a* negative and b* positive, the material tends to yellowness and redness colors. This effect is more significant in the b* axis with the increasing of curcumin, which was expected. In Table 2, the material CH15CUR000 has the same color characteristics a* and b* as the control; this indicates that the chitosan at 1.5% does not have any effect on these coordinates.

### 3.3. Water Contact Angle

**Figure 3:**
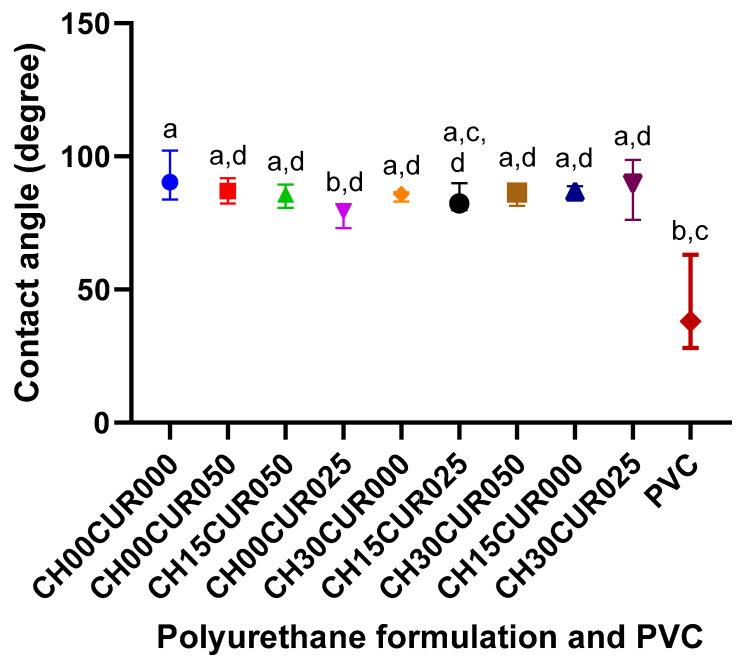
Contact angle of polyurethane formulations and PVC. Median with CI 95% of contact angle of polyurethane formulations and PVC. Any bar with the same letter is not significantly different (*p* > 0.05).

The water contact angle is used to measure the angle of a drop of water over the surface of a sample. It is used to establish whether a material is hydrophobic (contact angle higher than 90°) or hydrophilic (contact angle lower than 90°) [10]. Figure 3 shows that the polyurethane samples had a non-statistically significant difference with most of the polyurethane formulations; the only formulation with a statistically significant difference to CH00CUR000 is CH00CUR025. This could be due to curcumin at a 0.25% weight rate having more affinity with water, but at this amount, it shows lower physical interaction with the polyurethane matrix. As shown in Figure 3, the additives are dispersed on the polyurethane matrix (Figure 2); hence, the water does not interact with the additives, keeping a very similar contact angle among all the polyurethane samples. On the other hand, the PVC film showed the highest hydrophilicity with a statistically significant difference from most of the polyurethane formulations. Due to the polyurethane formulations being hydrophobic, they are candidates for food packaging because they repel the water, which could reduce the spoilage of food.

### 3.4. FTIR Studies of Polyurethane Films

**Figure 4:**
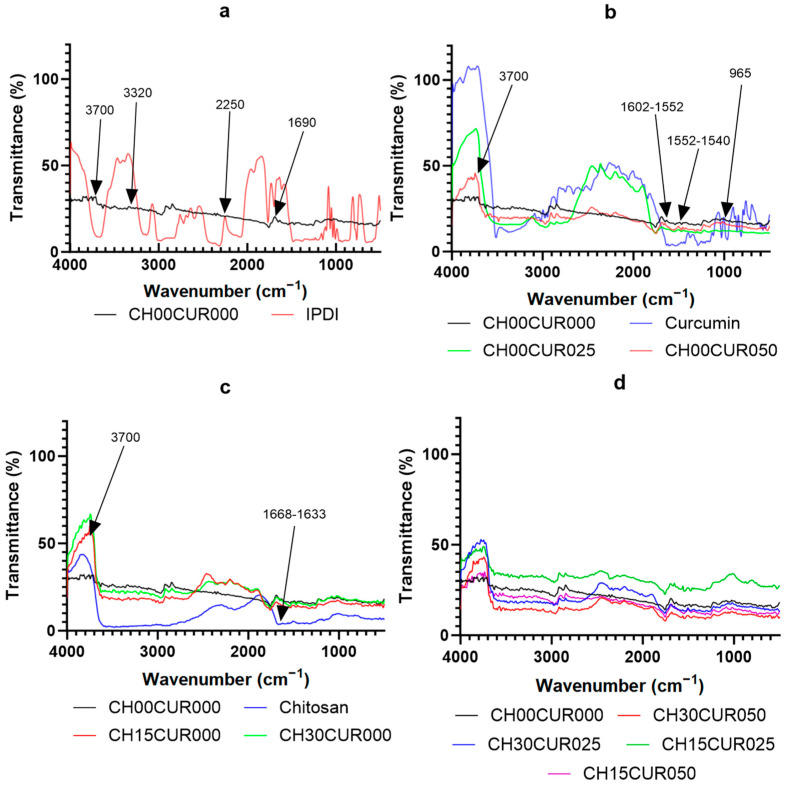
FTIR spectra of polyurethane formulations: (**a**) CH00CUR000 and isophorone diisocyanate (IPDI); (**b**) CH00CUR000, curcumin, CH00CUR025, CH00CUR050; (**c**) CH00CUR000, chitosan, CH15CUR000, CH30CUR000; (**d**) CH00CUR000, CH30CUR050, CH30CUR025, CH15CUR025, CH15CUR050.

FTIR analysis was carried out to compare the spectral differences of the polyurethane film formulations. The spectra are shown in Figure 4. The band in the 3320 cm^−1^ region is assigned to the stretching vibration of the N-H, and it confirms the presence of the 1690 cm^−1^ band of the C=O [53,54]. These N-H and C=O bands confirm the successful formation of polyurethane [54]. The 2250 cm^−1^ band of isocyanates disappeared. It is expected that the isocyanates and primary hydroxyl groups are totally consumed by the reaction [53,54]. There is a small peak at the 3700 cm^−1^ region [53], which is likely due to the low availability of the hydroxyl secondary groups of the castor oil polyol (Figure 4a).

We compared the FTIR spectra of the PU film without any additive against films containing only curcumin at 0.5% and 0.25% without chitosan. Figure 4b shows that in formulation CH00CUR050, there were no observed differences in the peaks of PU without additive CH00CUR000. This outcome could indicate that there is not any chemical interaction between the curcumin and polyurethane matrix. Likewise, PU formulation CH00CUR050 has a lower availability of hydroxyl groups than formulation CH00CUR025 due to the intensity of the peak at 3700 cm^−1^. This could infer that CH00CUR050 has a lower availability of hydroxyl groups due to the building of intermolecular forces, likely hydrogen bonds. This is a physical interaction of the additive with the PU polymer. The 1602 cm^−1^ band (benzene ring stretching vibrations) of curcumin is shifted to 1552 cm^−1^; the 1540 cm^−1^ band (C=O and C=C vibrations) of curcumin is shifted to 1468 cm^−1^; and the 965 cm^−1^ peak (C-O-C vibrations) of curcumin was smoother in the PU formulation [55]. It highlights that the likely formation of hydrogen bonds in the hydroxyl groups of curcumin even restricts the vibration stretching of the benzene ring. Then, these shifts could indicate that there are some physical interactions between curcumin and polymer.

Figure 4c shows that the formulations of polyurethane with chitosan and chitosan spectra. Since no new peaks are observed, it is possible to conclude that similarly to curcumin, chitosan strengthens polyurethane. CH30CUR00 and CH15CUR00 formulations show a very similar OH signature at 3700 cm^−1^ (Figure 4c). This could indicate that hydrogen bonds are built in the hydroxyl groups of chitosan at a very low intensity. The CONH_2_ (1668 cm^−1^) and NH_2_ (1633 cm^−1^) signatures of chitosan are not visible in both CH30CUR000 and CH15CUR000 formulations [56,57]. It is possible to shift at this band, but this is not observable due to the characteristic low peaks of the CONH_2_ and NH_2_ bonds. In contrast to the CH00CUR050 and CH00CUR025 formulations with several physical interactions between the additive and polyurethane, the formulations containing chitosan alone only show one interaction between chitosan and polyurethane (CONH_2_ and NH_2_ not observable shifts), showing a general weaker interaction.

**Scheme 2:**
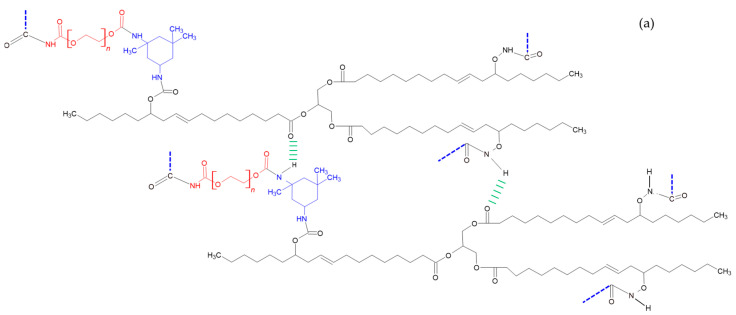

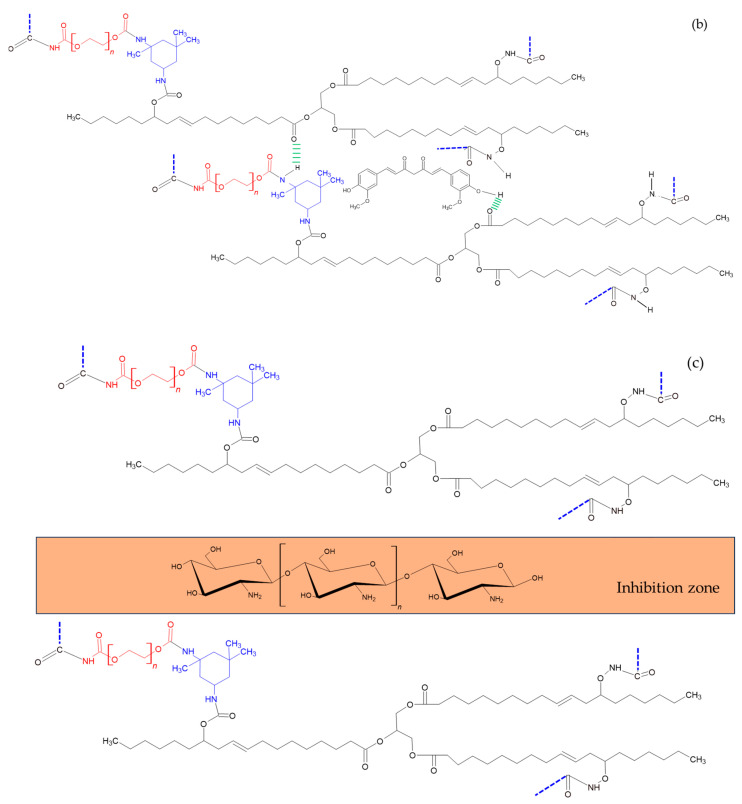
Illustration of some possible hydrogen bonds formation. (**a**) PU-PU, (**b**) PU-CUR-PU, (**c**) PU-CH. The hydrogen bonds are represented with the green horizontal lines.

Lastly, there are no important differences in the formulations combining chitosan and curcumin: CH15CUR050, CH15CUR025, CH30CUR05 and CH30CUR025. These formulations show a very similar behavior of assessing formulations with just one additive (chitosan or curcumin). This could indicate that there is not any interaction between chitosan and curcumin due to the low ratio of additives. Scheme 2 shows a diagram of the polyurethane–additives interactions.

### 3.5. Thermogravimetric Analysis (TGA) and Derivative Thermogravimetric Analysis (DGA)

**Figure 5:**
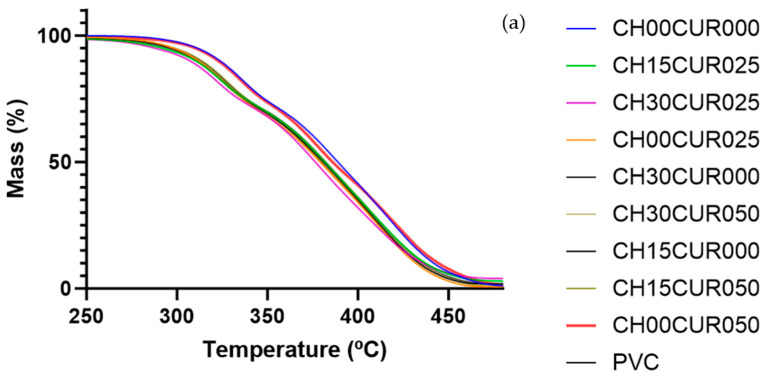

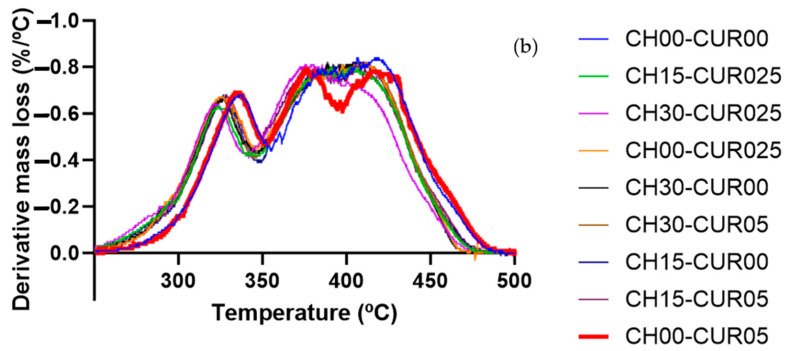
Thermogravimetric (TGA) (**a**) and derivative thermogravimetry (DGA) (**b**) curves of polyurethane formulations.

Figure 5a shows that the polyurethane thermograms were obtained from castor oil, IPDI and PEG. In this thermogram, it is possible to observe three regions. The first one is the degradation of PEG and minor components of castor oil. The second one is the degradation of urethane bonds of hard segments. The third one is the degradation of soft segments of polyurethane. Table 3 presents the onset temperature obtained from TGA, which is the temperature at which a 5% mass loss occurs. As seen in Table 3, the value of the onset temperature is higher for polyurethane without additives. Due to curcumin and chitosan being strengthening fillers, they are not part of the polyurethane matrix (the degradation temperature of chitosan and curcumin is lower than 300 °C). Among the polyurethanes containing both curcumin and/or chitosan, there is no significant difference, which validates the fact that there is not any chemical interaction between additives and the polyurethane matrix. For the polyurethanes containing chitosan and curcumin, we found the lowest onset temperature, which might indicate a little physical interaction between chitosan and curcumin. It highlights that increasing the ratio of curcumin when combining formulations improved the resistance against temperature due to there being better physical interactions. Figure 5b shows the DGA curves of the PU formulations. Overall, we observed a wide peak between 380 and 450 °C that corresponded to the second and third degradation zones, which was linked to the hard and soft segments’ polyurethane degradation. The fact that there were not different degradation zones among the polyurethane with or without additives indicates that there was not any chemical reaction between the additives and polyurethane. This displacement is caused by a weaker interaction between the additives and the polymer as well as the powder morphology of curcumin and chitosan. In contrast with the remaining materials, the PU formulation CH00CUR050 is the only one with three peaks of degradation. This result indicates the hydrogen bonding interactions among the polyurethane matrix and the curcumin through free hydroxyl groups (as it was mentioned in the FTIR section).

### 3.6. Dynamic Mechanical Thermal Analysis (DMTA)

**Figure 6:**
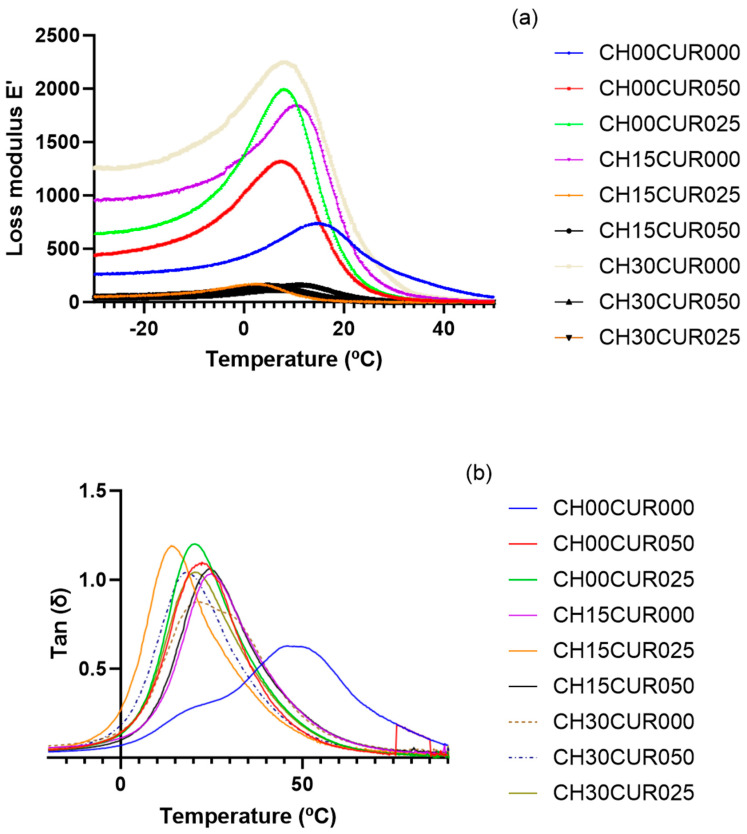
Dynamic mechanical thermal analysis (DMTA) of polyurethane formulations. (**a**) The loss modulus, (**b**) the Tan(δ).

DMTA assays in polymers measure the modulus (stiffness) and damping (energy dissipation) properties as a temperature function. The tan(δ) is a parameter that relates the modulus and damping of a polymer; through tan(δ), it is possible to establish the glass transition temperature (T_g_), compatibility of blends, and the effect of degree of cross-linking on viscoelastic properties, among other properties. Figure 6a shows the storage modulus of polyurethane with temperature. It can be seen that the materials containing chitosan or curcumin (but not both) have a higher storage modulus E′ compared to polyurethane without additives. This increase indicates that these materials have a higher rigidity than polyurethane without an additive. Adding an additive to the polyurethane matrix reduces the molecular movement of the chain because it decreases the free volume. In addition, the materials containing both chitosan and curcumin have a lower storage modulus compared to chitosan without additives. This reveals the low interaction between chitosan and curcumin due to thermodynamic incompatibility. Figure 6b shows a displacement of the tan(δ) to the left. This displacement indicates a decrease in the T_g_ temperature linked with the free volume of the molecules. This outcome suggests that after incorporating chitosan and curcumin, the motions of the main chain of polyurethane decrease. When additives are added, the intensity of the tan(δ) decreases due to the restriction of the motion chain increasing. These results validate that chitosan and curcumin are external agents to the polyurethane matrix. The reduction in free volume when the additives are incorporated indicates the motion inhibition of chains due to the presence of intermolecular forces like hydrogen bonding among additives and the polyurethane matrix.

According to Szycher [58], there are four types of intermolecular forces among polyurethane molecules, which here are ranked from strongest to weakest: ionic bonding, hydrogen bonding, dipole interaction and van der Waals forces. In terms of the chemical structure of polyurethane formulation in this research, the ionic and dipole bonding are discarded, while hydrogen bonding and van der Waals forces are the most probable interactions. However, despite being more frequent, the van der Waals forces are too weak. Therefore, the hydrogen bonding among molecules of polyurethanes is the strongest interaction [25]. Nevertheless, in line with FTIR and TGA findings, it is possible that the additives physically interact with a molecule of polyurethane, causing hydrogen bonding to occur between the additive and a single molecule of polyurethane instead of among molecules of polyurethane. This could explain why the free volume increases with the addition of curcumin and/or chitosan. Another possible reason is that chitosan is a large molecule, which occupies the space between polyurethane molecules, blocking the formation of hydrogen bonding among polyurethanes.

### 3.7. Mechanical Properties

Table 3 shows the outcomes for mechanical properties of polyurethane formulations. When it aggregates one of two additives (not both simultaneously), the Young’s modulus and tensile strength increase, but elongation at break decreases. When only one additive is incorporated, the rigidity of the material increases. It is possible that each additive individually is more compatible with the polyurethane matrix due to the formation of hydrogen bolding compared to the incorporation of chitosan and curcumin at the same time. This is an inhibition effect: when chitosan and curcumin are present, the interactions between polyurethane and additives decrease. In contrast, materials containing curcumin and chitosan have the lowest mechanical properties. This is coherent with the obtained results of DMTA analysis.

### 3.8. Differential Scanning Calorimetry (DSC)

**Figure 7:**
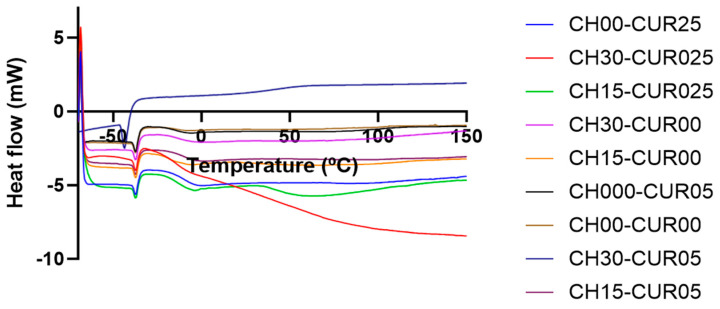
Differential scanning calorimetry (DSC) of polyurethane formulations.

In Figure 7, it is possible to observe that all the polyurethane samples have an amorphous structure. Additionally, the sample CH15CUR025 shows two phases, which means a higher incompatibility. This is consistent with DMTA results, where this sample had the lowest T_g_.

### 3.9. Overall and Specific Migration

Overall migration is an assay to test how many masses could migrate from a material in contact with food to the produce. Thus, migration is a mass transfer phenomenon influenced by kinetics and thermodynamics. These migrants move through empty spaces and other breaks among molecules; thus, migration depends on the migrant’s properties and the size and structure of the empty spaces [59]. According to Colombian legislation, a material in contact with food does not be more than 50 mg kg^−1^ (milligrams of material in contact with food over kilograms of produce) [2]. For the European Union legislation, it is 60 mg kg^−1^ [3].

**Figure 8:**
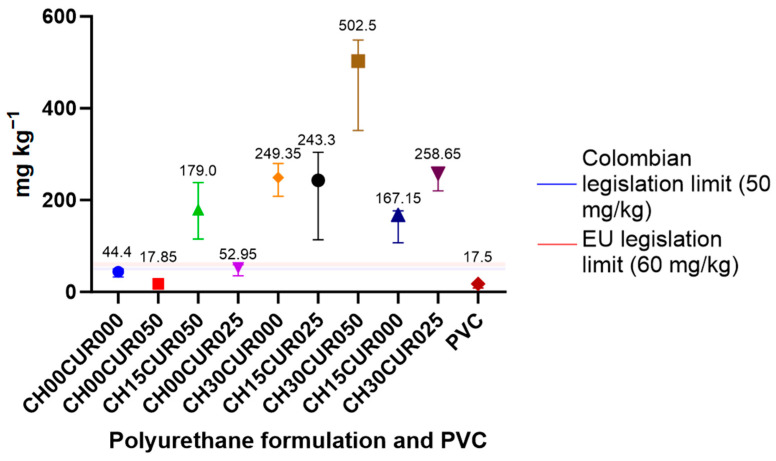
Median with CI 95% of overall migration of polyurethane formulations and PVC. The significantly different groups (*p* < 0.05) were CH00CUR000-CH30CUR050, CH00CUR050-CH30CUR050 and CH30CUR050-PVC.

Figure 8 shows the median of the obtained data. The formulation without additives (CH00CUR000) had a median below 50 mg/kg. The formulations containing chitosan overcome 50 mg/kg in all cases, whereas formulations containing only curcumin (CH00CUR025 and CH00CUR050) had lower values.

We performed the specific migration of isophorone diisocyanate (IPDI), chitosan and curcumin. Due to the high toxicity of IPDI [60], we measured the specific migration to test the safety of the polyurethane formulations for food packaging. Chitosan and curcumin were performed to probe the assumption that the overall migration mass consists of mostly additives.

The outcomes of specific migration are shown in Table 3. The specific migration of IPDI was found to be lower than 3.85 mg/kg, indicating that the migration could be caused by unreacted IPDI from the polyurethane chemical reaction. On the other hand, the specific migration of curcumin was found to be very low and was only detected in the formulation CH30CUR050. Chitosan-specific migration values were detected in five out of nine formulations. The chitosan migration increased when it increased the ratio of chitosan on the formulation. The remaining two formulations where chitosan was detected were CH15CUR025 and CH15CUR000. The obtained results suggest that there is a low migration for IPDI, chitosan and curcumin. In the case of polyurethane films containing curcumin and chitosan, it is possible to infer that despite the fact that they both are incompatible, they stay captured on the polyurethane matrix. It was observed that additives and IPDI just explain a little part of the migrated mass. Then, we determined the remaining molecular masses of the lasting residues of overall migration. We found that a great amount of all the PU samples had a high percentage of migrated oligomers (in Table 4, results are reported as the ratio of oligomers): from 16.19% to 74.31%. Considering these results, the residue was analyzed through UPLC QTOF. The results suggest that the migrated mass PEG chains were of low molecular weight.

The migration rate of a material in contact with food depends on the packaging material, the type of contact (direct or indirect), the kind of food, its solubility, the initial concentration of migrant in the material, its structure, its molecular size and its polarity [61,62,63]. Overall, the migration of additives hinges on their molecular weight; the higher the molecular size, the lower its migration [64]. However, this assumption ignores the intermolecular forces built among additive–polymer interactions and the inhibition of intermolecular forces among polymer–polymer molecules. Contrary to most of the scientific literature, in this research, it was evidenced that the polyurethane formulation with the additive that had the highest molecular weight (chitosan) had a great influence on increasing the overall migration. According to specific migration outcomes, there was a very low migration of additives compared to total mass. Then, according to the analysis of molecular weights of overall migration, the specific migration could be explained by oligomers or polymers of low molecular weights. On the other hand, the curcumin was supposed to have higher migration values considering its low molecular weight; however, it showed the lowest migration values. This could be explained by the building of hydrogen bonds among curcumin–polyurethane, as shown in the aforementioned results. Turan and Gunes [23] assessed the overall migration on a film of polyurethane without additives; they obtained 7.2 mg dm^−2^, while in this research, the formulation without additives was 17.18 mg dm^−2^.

According to Chen et al. [65], when chitosan is the only polymeric matrix, the migration mass of materials with functionalized chitosan is relatively low in comparison to other migration rates. However, when chitosan is blended with other polymers, the migration rates tend to increase. Winotapun et al. [66] assessed the overall migration of a polymer blended with chitosan and lignin; when the chitosan ratio in the blend was above 89%, the migration values were below 5 mg dm^−2^, but when the chitosan ratio was lower than 80%, the migration rates increased to higher values than 22.5 mg dm^−2^. Similarly, Merino et al. [67] studied different formulations of polymers; when they assessed chitosan at its highest ratio (70%), this increased the overall migration to a statistically significant difference compared to the remaining formulations. These results are in concordance with the findings of the current study; the chitosan seems to be incompatible with most of the other polymers, which increases the overall migration.

On the other hand, Gülay and Faik [68] used a matrix of starch, using curcumin as one of the additives, and they obtained an overall migration of 5.78 mg dm^−2^. This finding is in concordance with the results of this research; however, different formulations and specific migration were not discussed by Baysal and Doğan [68]. Likewise, Ma et al. [69] found that curcumin weakened the tara gum or polyvinyl alcohol intermolecular forces; this outcome does not in concordance with the findings of the present research. It is possible that curcumin had a different performance and compatibility depending on the polymer matrix.

It is necessary to conduct further research to establish the specific migration of the remaining non-identified compounds, as there was migration that was not explained by isophorone diisocyanate, chitosan or curcumin. It highlights that there are limitations to identifying migrated compounds, since it is possible that some of them are polymers of different molecular weights, and it is difficult to identify the molecule. This is because HPLC is the main technique to identify specific migration, but previously, it required having a sample of high purity and knowing the molecular weight of the compound of interest.

Migration is one of the most important areas to establish the safety of a material intended for food packaging. According to the inventory of food contact substances by the U.S. Food and Drug Administration, there are just a few polyurethane formulations approved for use in food packaging [70]. It is possible that the use of isocyanates to synthesize polyurethanes discourages the polyurethanes for food packaging due to the usually high toxicity of isocyanates [60] despite polyurethanes being used for biomaterials (materials designed to be in contact with human tissues) [8]. Thus, it is necessary to conduct further research on polyurethanes for food packaging, mainly assessing their toxicity, in order to provide more evidence of the safety of polyurethanes for food packaging.

### 3.10. Water Vapor Transmission Rate (WVTR)

The WVTR outcomes are shown in Table 3. The results showed that PVC had a statistically significant higher WVTR than any polyurethane films obtained in this work. There was no statistically significant difference among polyurethane formulations. This is caused by the superficial hydrophobic of the polyurethane films, which is evidenced by the contact angle results (Figure 3). We carried out a coefficient of determination (r^2^) test and found a r^2^ of 0.502, which indicates that the thickness of the films was not a factor that could affect WVTR results. The low WVTR values obtained for polyurethane films are desirable for many fruits and vegetables packaging to reduce the wet environment that allows colonization by microbes. The water vapor transmission rate of the polyurethane films of the present research showed lower values than the PUs formulation of Turan [25]. There are two possible explanations for this difference: the hydrophobic performance of the polyurethane films of the present research, and the high ratio of castor oil on the formulation, since according to Turan, increasing castor oil “may reduce sorption of polyurethane films”.

### 3.11. Oxygen Transmission Rate (OTR)

The outcomes of all nine formulations and PVC film showed a high permeation of oxygen, which was out of the limit of equipment detection (*x* > 200 cc/m^2^.day). This is caused by the amorphous structure of polyurethane (evidenced by the DSC results, (Figure 7)); this structure leaves voids that are used by oxygen to pass through the film [71]. On the other hand, barrier properties outcomes showed similar results to the scientific literature [25] with low values of water vapor transmission rate. Additionally, the polyurethane films were shown to be permeable to the oxygen transmission rate due to their amorphous structure.

## 4. Conclusions

Polyurethane food packaging with chitosan and curcumin as additives was analyzed. The additives are dispersed in the polyurethane matrix with physical interaction rather than chemical interactions with polyurethane, which took place likely through hydrogen bonding. These possible physical interactions led to important differences in the mechanical performance and overall migration. Where there was a lower physical interaction through hydrogen bonding, the elongation at break was higher due to the better mobility among polymers. In the same way, the polyurethane formulations with chitosan had the highest overall migration due to the low compatibility among chitosan and polyurethane as well as the low physical interactions among them. The curcumin and polyurethane physical interactions showed a better compatibility, which reduced the overall migration, despite curcumin having lower molecular weight than chitosan. According to the obtained results, we recommend the two formulations CH00CUR000 and CH00CUR050 for food packaging films, because they both show under 50 mg kg^−1^ of overall migration, which is required for Colombian legislation. The mentioned polyurethane films had comparable performance with PVC films in overall migration, but overcoming the mechanical performance of PVC is still a challenge.

## Figures and Tables

**Table 1:** Polyurethane formulations and codes.

Run	Code	Formulation
1	CH00CUR000	Chitosan 0%, curcumin 0%
2	CH00CUR050	Chitosan 0%, curcumin 0.5%
3	CH15CUR050	Chitosan 1.5%, curcumin 0.5%
4	CH00CUR025	Chitosan 0%, curcumin 0.25%
5	CH30CUR000	Chitosan 3.0%, curcumin 0%
6	CH15CUR025	Chitosan 1.5%, curcumin 0.25%
7	CH30CUR050	Chitosan 3.0%, curcumin 0.5%
8	CH15CUR000	Chitosan 1.5%, curcumin 0%
9	CH30CUR025	Chitosan 3%, curcumin 0.25%

**Table 2:** Color measurements of polyurethane films.

Polyurethane Formulation	L*	a*	b*
CH00CUR000	89.13 ± 1.29 ^a^	−0.93 ± 0.04 ^a^	6.79 ± 0.17 ^a^
CH00CUR050	83.84 ± 0.62 ^a^	−12.29 ± 0.16 ^b^	50.92 ± 0.45 ^b^
CH15CUR050	84.32 ± 0.16 ^a^	−14.34 ± 0.08 ^b^	47.30 ± 0.09 ^b^
CH00CUR025	86.96 ± 0.90 ^a^	−15.71 ± 0.99 ^b^	44.29 ± 2.49 ^b^
CH30CUR000	81.22 ± 1.78 ^b^	−4.69 ± 0.12 ^b^	22.12 ± 0.56 ^b^
CH15CUR025	89.66 ± 0.59 ^a^	−16.56 ± 0.20 ^b^	41.24 ± 1.23 ^b^
CH30CUR050	87.85 ± 1.03 ^a^	−15.41 ± 0.29 ^b^	44.72 ± 0.63 ^b^
CH15CUR000	88.79 ± 0.80 ^a^	−0.47 ± 0.06 ^a^	6.18 ± 0.25 ^a^
CH30CUR025	85.02 ± 0.73 ^a^	−15.51 ± 0.12 ^b^	41.21 ± 0.14 ^b^

The same letter means there are no statistically significative differences between control CH00CUR000 and remaining groups (*p* < 0.05).

**Table 3:** Mechanical properties, onset temperature and water vapor transmission rate (WVTR) of polyurethane formulations and commercial PVC film with standard deviation.

Polyurethane Formulation	Young’s Modulus (MPa)	Tensile Strength (Mpa)	Elongation at Break (%)	Onset Temperature (°C)	WVTR (g/Pa.s.m^2^)
CH00CUR000	1.79 ± 0.18 ^a^	1.1 ± 0.08 ^a^	101.94 ± 28.49 ^a^	311.5	1.868 × 10^−7^ ± 7.496 × 10^−8^
CH00CUR050	2.17 ± 0.15 ^a^	1.2 ± 0.13 ^b^	77.48 ± 10.34 ^a^	310.17	2.442 × 10^−7^ ± 9.048× 10^−8 a^
CH15CUR050	2.45 ± 0.19 ^a^	1.6 ± 0.15 ^c^	118.83 ± 18.30 ^a^	298.67	1.706 × 10^−7^ ± 3.326 × 10^−8 a^
CH00CUR025	2.50 ± 0.09 ^a^	1.8 ± 0.19 ^d^	125.50 ± 26.72 ^a^	298.00	1.816 × 10^−7^ ± 4.594 × 10^−8 a^
CH30CUR000	2.02 ± 0.14 ^a^	1.4 ± 0.16 ^a^	112.55 ± 18.71 ^a^	296.33	2.202 × 10^−7^ ± 6.912 × 10^−8 a^
CH15CUR025	1.57 ± 0.12 ^a^	0.9 ± 0.09 ^a^	95.47 ± 16.18 ^a^	292.33	2.319 × 10^−7^ ± 4.05 × 10^−8 a^
CH30CUR050	2.12 ± 0.25 ^a^	1.4 ± 0.19 ^a^	107.61 ± 14.11 ^a^	297.17	2.403 × 10^−7^ ± 1.136 × 10^−7 a^
CH15CUR000	1.66 ± 0.09 ^b^	1.3 ± 0.15 ^e^	163.89 ± 32.85 ^b^	297.00	1.347 × 10^−7^ ± 5.521 × 10^−8 a^
CH30CUR025	1.31 ± 0.10 ^a^	0.8 ± 0.13 ^a^	96.68 ± 24.15 ^a^	288.17	1.823 × 10^−7^ ± 7.106 × 10^−8 a^
PVC	147.64 ± 5.2 ^c^	29.78 ± 0.25 ^f^	185.43 ± 12.16 ^a^	240.8	9.817 × 10^−7^ ± 8.283 × 10^−8 b^

Any same letter means there are not statistically significant differences among the polymer formulations compared to control (CH00CUR000) (*p* > 0.05) with the exception of WVTR, where all groups were compared (*p* > 0.05).

**Table 4:** Specific migration of isophorone diisocyanate (IPDI), curcumin and chitosan of polyurethane formulations.

Polyurethane Formulation	IPDI (mg/kg)	Curcumin (mg/kg)	Chitosan (mg/kg)	Percentage of Monomer Over Overall Migration
CH00CUR000	3.36	Out of minimum detection (*x* < 0.09)	Out of minimum detection (*x* < 0.01)	Monomer (74.31%)
CH00CUR050	2.17	Out of minimum detection (*x* < 0.09)	Out of minimum detection (*x* < 0.01)	Monomer I (19.3%) Monomer II (15.96%)
CH15CUR050	3.83	Out of minimum detection (*x* < 0.09)	Out of minimum detection (*x* < 0.01)	Monomer I (11.18%) Monomer II (25.23)
CH00CUR025	2.00	Out of minimum detection (*x* < 0.09)	Out of minimum detection (*x* < 0.01)	Monomer I (30.59%) Monomer II (7.33%)
CH30CUR000	2.73	Out of minimum detection (*x* < 0.09)	0.1	Monomer I (42.97%) Monomer II (8.08%)
CH15CUR025	2.46	Out of minimum detection (*x* < 0.09)	0.19	Monomer I (33.10%) Monomer II (13.9%)
CH30CUR050	3.85	0.10	0.02	Monomer I (32.52%) Monomer II (14.41%)
CH15CUR000	2.85	Out of minimum detection (*x* < 0.09)	0.03	Monomer I (21.39%) Monomer II (15.17%)
CH30CUR025	3.34	Out of minimum detection (*x* < 0.09)	0.11	Monomer I (6.24%) Monomer II (9.95%)

## Data Availability

Data are contained within the article.
